# Integration of GWAS and eQTL Analysis to Identify Risk Loci and Susceptibility Genes for Gastric Cancer

**DOI:** 10.3389/fgene.2020.00679

**Published:** 2020-07-10

**Authors:** Jing Ni, Bin Deng, Meng Zhu, Yuzhuo Wang, Caiwang Yan, Tianpei Wang, Yaqian Liu, Gang Li, Yanbing Ding, Guangfu Jin

**Affiliations:** ^1^Department of Epidemiology, Center for Global Health, School of Public Health, Nanjing Medical University, Nanjing, China; ^2^Jiangsu Key Lab of Cancer Biomarkers, Prevention and Treatment, Collaborative Innovation Center for Cancer Medicine, Nanjing Medical University, Nanjing, China; ^3^Department of Gastroenterology, Affiliated Hospital of Yangzhou University, Yangzhou, China; ^4^Department of General Surgery, Jiangsu Institute of Cancer Research, Jiangsu Cancer Hospital, The Affiliated Cancer Hospital of Nanjing Medical University, Nanjing, China

**Keywords:** gastric cancer, eSNP, genome-wide association study, gene-based analysis, Sherlock integrative analysis

## Abstract

Genome-wide association studies (GWAS) have identified several susceptibility loci for gastric cancer (GC), but the majority of identified single-nucleotide polymorphisms (SNPs) fall within the non-coding region and are likely to exert their biological function by modulating gene expression. To systematically estimate expression-associated SNPs (eSNPs) that confer genetic predisposition to GC, we evaluated the associations of 314,203 stomach tissue-specific eSNPs with GC risk in three GWAS datasets (2,631 cases and 4,373 controls). Subsequently, we conducted a gene-based analysis to calculate the cumulative effect of eSNPs through sequence kernel association combined test and Sherlock integrative analysis. At the SNP-level, we identified two novel variants (rs836545 at 7p22.1 and rs1892252 at 6p22.2) associated with GC risk. The risk allele carriers of rs836545-T and rs1892252-G exhibited higher expression levels of *DAGLB* (*P* = 3.70 × 10^–18^) and *BTN3A2* (*P* = 3.20 × 10^–5^), respectively. Gene-based analyses identified *DAGLB* and *FBXO43* as novel susceptibility genes for GC. *DAGLB* and *FBXO43* were significantly overexpressed in GC tissues than in their adjacent tissues (*P* = 5.59 × 10^–7^ and *P* = 3.90 × 10^–6^, respectively), and high expression level of these two genes was associated with an unfavorable prognosis of GC patients (*P* = 1.30 × 10^–7^ and *P* = 7.60 × 10^–3^, respectively). Co-expression genes with these two novel genes in normal stomach tissues were significantly enriched in several cancer-related pathways, including P53, MAPK and TGF-beta pathways. In summary, our findings confirm the importance of eSNPs in dissecting the genetic basis of GC, and the identified eSNPs and relevant genes will provide new insight into the genetic and biological basis for the mechanism of GC development.

## Introduction

Gastric cancer (GC) is the fifth most common neoplasm and second leading cause of cancer-related death globally. There were approximately one million newly diagnosed GC cases and 780,000 deaths in 2018 (Bray et al., 2018). Approximately half of the new GC cases and deaths worldwide occur in China, indicating a major public health burden (Chen et al., 2016). A large twin cohort study in Nordic countries suggested that up to 22% interindividual variability in GC risk could be explained by genetic factors (Mucci et al., 2016). In the past decade, we and other groups have reported a number of susceptibility loci for GC through genome-wide association study (GWAS), which only explain a fraction of GC heritability (Abnet et al., 2010; Shi et al., 2011; Wang et al., 2017; Park et al., 2019). Moreover, the vast majority of disease-related variants discovered by GWAS fall within intergenic or non-coding regions, which may regulate the expression of target genes and influence the process of pathogenesis (Maurano et al., 2012).

Expression quantitative trait locus (eQTL) analysis has been conducted to provide prior weights for the statistical analysis of new susceptibility single-nucleotide polymorphism (SNP) discovery and prioritize SNPs or genes for further functional experiments (Li et al., 2013). Integration of GWAS and eQTL can help us dissect genetic mechanism of multiple diseases (Guo et al., 2018; Heinrichs et al., 2018). The Genotype-Tissue Expression (GTEx) project has established the largest comprehensive public database with whole-genome and transcriptome sequencing data across 53 normal human tissues from nearly 1,000 individuals, making it better to dissect the effects and molecular mechanism of functional variations.

In a given gene, several variants modulate its expression level in stomach tissue. These expression-associated SNPs (eSNPs) may synergistically regulate the expression of the target gene. Thus, collections of multiple genetic variants, rather than individual highly significantly associated eSNPs, may account for the putative role of the novel gene in predisposition to GC. Pathway-based analysis evaluates the cumulative effect of multiple SNPs from the same gene set. Utilizing this approach, several novel genes and biological pathways enriched with significantly disease-associated SNPs were identified (Cheng et al., 2016; Yao et al., 2016; Walsh et al., 2019). Generally, most studies select the representative SNPs by their proximity to a specific gene, which inevitably obscures the genetic effect between the candidate gene and disease. Accordingly, incorporating functional eSNPs into the pathway analysis is appealing because of its ability to explore the mechanism of complex diseases. Through evaluating the cumulative effect of 322,324 eSNPs in Caucasian individuals, scientists found that the autoimmune thyroid disease pathway and JAK-STAT pathway were involved in basal cell carcinoma pathogenesis (Zhang et al., 2012). Moreover, a similar strategy was also applied to obtain biological insight into the development of lung cancer and type 2 diabetes (Zhong et al., 2010; Wang et al., 2018). During the preparation of the manuscript, another similar computational method called loci2path was reported (Xu et al., 2020).

Considering the fact that regulatory causal variants confer to GC risk by affecting their target gene expression, we initially conducted genome-wide screening of 389,207 potential eSNPs in stomach tissues from the GTEx database. We then evaluated the associations of 314,203 eSNPs shared in three GWAS datasets with GC risk. In addition, we performed a gene-based analysis to calculate the cumulative effect of eSNPs and identify additional susceptibility genes that might help provide new insight into the mechanism of GC.

## Materials and Methods

### eSNP Analysis

Expression-associated SNPs in stomach tissues were derived from the GTEx v7 database (Stomach.allpairs.txt.gz). Genotyping was performed using Illumina HumanOmni 5 M and 2.5 M. Transcriptome dataset was generated by Affymetrix Expression Array or Illumina TruSeq RNA sequencing. A total of 237 stomach tissues with both genotype and expression data were available. Linear regression analysis was applied to evaluate the association between genetic variants and expression levels of genes within 1 Mb distance. As a result, a total of 636,426 *cis*-eQTL gene (eGene) pairs were defined with a false discovery rate (FDR) *P*-value < 0.05. After excluding indels, duplicated and non-biallelic eSNPs, there were 389,207 eSNPs remained.

### GC GWAS Datasets

Three existing GC GWAS datasets were used in the current study, including 2,631 cases and 4,373 controls. Of them, NJ-GWAS, and BJ-GWAS were previously conducted by our group (Shi et al., 2011). All subjects recruited from Nanjing (550 cases and 1,155 controls) and Beijing (456 cases and 1,118 controls) were genotyped with Affymetrix Genome-Wide Human SNP Array 6.0. Another GC GWAS dataset named SX-GWAS was approved and downloaded from the dbGap (accession number: phs000361.v1.p1; Abnet et al., 2010). All participants (1,625 cases and 2,100 cancer-free individuals) recruited from Shanxi and Linxian were genotype using the Illumina 660W-Quad chips. The basic characteristics of study participates were shown in Supplementary Table S1.

### Quality Control and Imputation for GWAS

We performed a standard quality control procedure for these three GWAS by excluding samples with lower call rates, sex discordance, or excessive heterozygosity. Then, we excluded eSNPs with a call rate < 95%, minor allele frequency (MAF) <0.01, or *P* < 1 × 10^–6^ for Hardy-Weinberg equilibrium. Imputation was performed with SHAPEIT v2 (Delaneau et al., 2011) and IMPUTE2 (Howie et al., 2009) with the 1000 Genomes Project (Phase III integrated variant set release, across 2,504 samples) as reference. We selected eSNPs with INFO score ≥ 0.4 for further association analysis.

### Association Analysis

For each eSNP, unconditional logistic regression was conducted to calculate odds ratios (ORs), and 95% confidence intervals (CIs). We performed genetic association analysis assuming an additive effect model with adjustment for age, sex, smoking, alcohol consumption, and top ten principal components (PCs) in NJ-GWAS and BJ-GWAS. Since the smoking and drinking status were not available in the SX-GWAS dataset, we took age, sex and top ten PCs as covariates. Subsequently, a meta-analysis with the fixed-effects model was conducted to pool the results from each GWAS by using the GWAMA software (Magi and Morris, 2010). *I*^2^ indicates the percentage of the effect estimates variability which can be attributed to heterogeneity, and an *I*^2^ value of ≥75% represents high heterogeneity. We filtered significant eSNPs on linkage disequilibrium (LD; *r*^2^ < 0.1), from which, the index eSNPs with the lowest *p* value in each LD block were obtained. All statistical analyses were conducted by using PLINK 1.9 and R language (version 3.5.0). Regional association plots were generated in LocusZoom.

### Variance Explained

The phenotypic variance explained by genetic variants was estimated using the fixed-effects model in the single-variant analysis as previously described (Lee et al., 2012). Variants identified in the present study and those published in previous GWAS (Supplementary Table S2) were used to calculate the respective variances by assuming the 5-year prevalence of GC to be 32.43/100,000, 42.43/100,000, and 52.43/100,000 in China^1^.

### *In silico* Functional Annotation

We used ANNOVAR (Wang et al., 2010) to generate gene-based annotation and then described the distribution of all these eSNPs. We extracted candidate SNPs in strong LD (*r*^2^ ≥ 0.6) with the index variant based on the 1000 Genomes Phase 1 Asian individuals from the online HaploReg v4.2 tool (Ward and Kellis, 2012). According to the available data from ENCODE (Ward and Kellis, 2012) and Roadmap (Bernstein et al., 2010) we predicted regulatory elements (promoter, enhancer, etc.) through histone modification markers (H3K4me3, H3K4me1, and H3K27ac) and chromatin state segmentation in the stomach tissues and DNase I hypersensitivity sites (DHS) in 125 cell types. Other bioinformatics annotation tools, including RegulomeDB (Supplementary Table S3; Boyle et al., 2012) CADD (Kircher et al., 2014) GWAVA (Ritchie et al., 2014) and PINES (Corneliu et al., 2018) were also used to decipher the potential functional variants.

### Gene-Based and Pathway Analysis

Gene-based analysis was performed using the sequence kernel association combined test (SKAT-C), which calculates the combined effect of common variants toward a particular phenotype (Ionita-Laza et al., 2013). Pathway analysis was conducted in merged dataset by the adaptive rank truncated product (ARTP) method with 10,000 permutations, which utilizes highly efficient permutations to analyze the association between genes within a pathway and diseases (Yu et al., 2009). All analyses were implemented in R package “SKAT” and “ARTP.” Human-derived gene sets were cataloged by and obtained from the Molecular Signatures Database (MSigDB, version 6.2). Finally, a total of 1,077 pathways with 5,155 related genes were derived from KEGG (*n* = 186), Reactome (*n* = 674), and BioCarta (*n* = 217). The Benjamini-Hochberg method was applied to correct multiple testing, setting the threshold for significance at 5% FDR. In addition, genes were considered significant when they had *P*-values < 0.05 in at least two GWAS datasets.

### Sherlock Integrative Analysis

We used Sherlock integrative analysis for further validation (He et al., 2013). Sherlock uses a Bayesian statistical method to calculate the individual Bayes factor for each eSNP, and their sum constitutes the final Log Bayes factor (LBF) score for each gene. The larger LBF score represents the higher probability that the gene is associated with GC. If an eSNP is significantly associated with GC, a positive score would be assigned. Otherwise, a negative LBF score would be given. The *P* threshold for statistical significance was set to 1.0 × 10^–3^.

### Differential Expression Analysis

We downloaded the normalized expression data and clinical information of individuals with GC from The Cancer Genome Atlas database. Differential expression analyses were performed in 32 paired gastric tumor and adjacent normal tissues.

### Co-expression and Gene-Set Enrichment Analysis

The expression data of 23,424 genes in 237 normal stomach tissues were obtained from the GTEx v7 database. We conducted genome-wide expression correlation analysis to identify co-expression genes with the linear regression model. Gene-set enrichment analysis (GSEA) of the KEGG pathway gene set collection was implemented in R package “clusterProfiler” (Yu et al., 2012). All genes were pre-ranked according to the Pearson correlation coefficients calculated by the co-expression analysis. Then, gene sets were considered significantly enriched if the FDR was <0.05 after 100,000 permutations.

## Results

### Individual eSNP Associated With GC Risk

**FIGURE 1:**
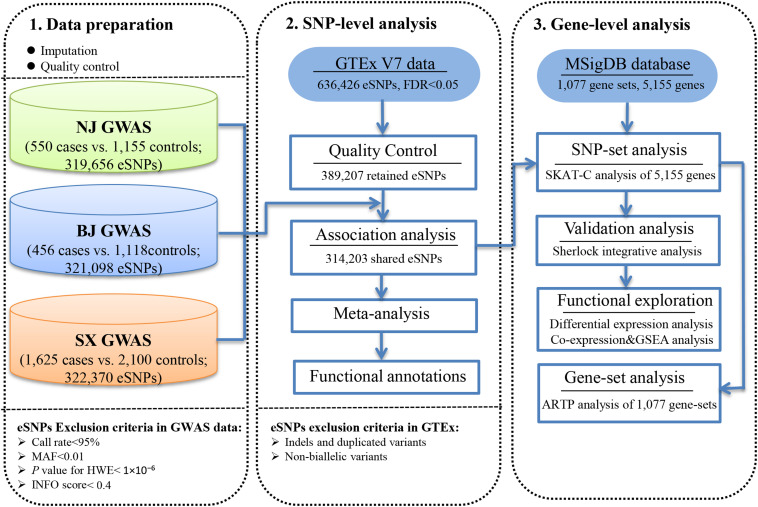
Workflow of the study design.

**FIGURE 2:**
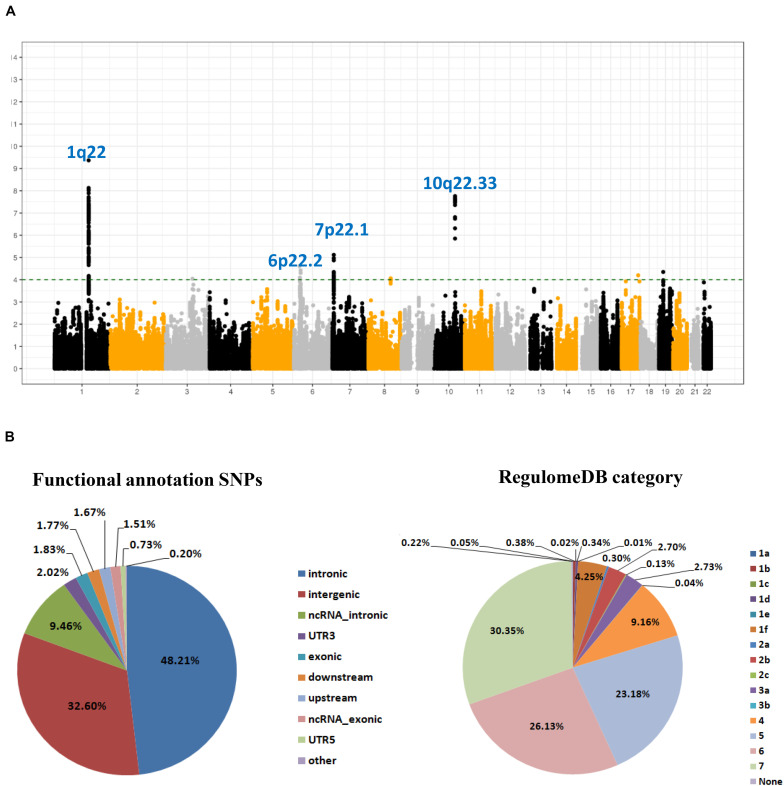
SNP-based associations with GC in the GWAS meta-analysis. **(A)** Manhattan plot of *P* value for each expression-related SNPs (eSNPs) highlighting key chromosomal regions. The associations [-log_10_ (*P*) values, *Y*-axis] are plotted against genomic position (*X*-axis by chromosome and the chromosomal position of NCBI build 37). The green horizontal line corresponds to a *P* value threshold of 1.00 × 10^–4^; **(B)** Pie charts showing the distribution of functional annotation and Regulome DB score (a categorical sore range from 1a to 7, indicating biological indicating biological evidence of a SNP being a regulatory element, with a low score denoting a higher likelihood of a SNP being regulatory) for 307,676 eSNPs without heterogeneity between studies.

As shown in the workflow chart (Figure 1), 389,207 eSNPs were found to be significantly associated with their surrounding gene expression levels (FDR < 0.05) in 237 stomach tissue samples from the GTEx database. Among them, 319,656, 321,098, and 322,370 eSNPs passed the quality control in NJ-GWAS, BJ-GWAS, and SX-GWAS, respectively. A total of 314,203 shared eSNPs were included in the genetic association analysis, and the association results of 307,676 variants without heterogeneity between studies (*I*^2^ < 75.0%) were shown in Figure 2A. Most of the eSNPs were located within intronic (48.21%) or intergenic (32.60%), and 8.19% had a RegulomeDB score less than 3 (Figure 2B). After LD pruning, we identified a total of 1,222 index eSNPs at *P* < 0.05. Among them, 4 eSNPs were retained after multiple testing correction (FDR < 0.05; Table 1). Region plots of these four significant variants were depicted in Supplementary Figure S1.

**TABLE 1:** Associations of four significant expression-related SNPs (eSNPs) with GC risk under the additive genetic model.

**SNP**	**Region**	**Alleles^a^**	**NJ-GWAS**	**BJ-GWAS**	**SX-GWAS**	**Fixed-effect meta-analysis**		
			**OR(95% CI)^b^**	**OR(95% CI)^b^**	**OR(95% CI)^c^**	**OR(95% CI)**	***P* value**	**FDR^d^**
rs6676150	1q22	G/C	0.67 (0.52–0.86)	0.79 (0.54–1.17)	0.55 (0.65–0.76)	0.67 (0.59–0.76)	4.29 × 10^–10^	3.41 × 10^–6^
rs12217597	10q23.33	T/C	1.05 (0.85–1.29)	1.31 (0.95–1.81)	1.28 (1.45–1.64)	1.33 (1.21–1.47)	1.74 × 10^–8^	6.92 × 10^–2^
rs836545	7p22.1	C/T	1.10 (0.91–1.33)	1.37 (1.04–1.81)	1.13 (1.26–1.41)	1.23 (1.12–1.35)	7.64 × 10^–6^	2.03 × 10^–2^
rs1892252	6p22.2	C/G	1.69 (1.33–2.16)	1.60 (1.05–2.43)	0.70 (0.89–1.14)	1.41 (1.20–1.66)	2.43 × 10^–5^	4.83 × 10^–2^

*^*a*^Reference allele/effect allele. ^*b*^Adjusted for age, gender, smoking, drinking and top ten principal components (PCs). ^*c*^Adjusted for age, gender and top ten PCs. ^*d*^FDR was corrected by Benjamini-Hochberg procedure.*

**FIGURE 3:**
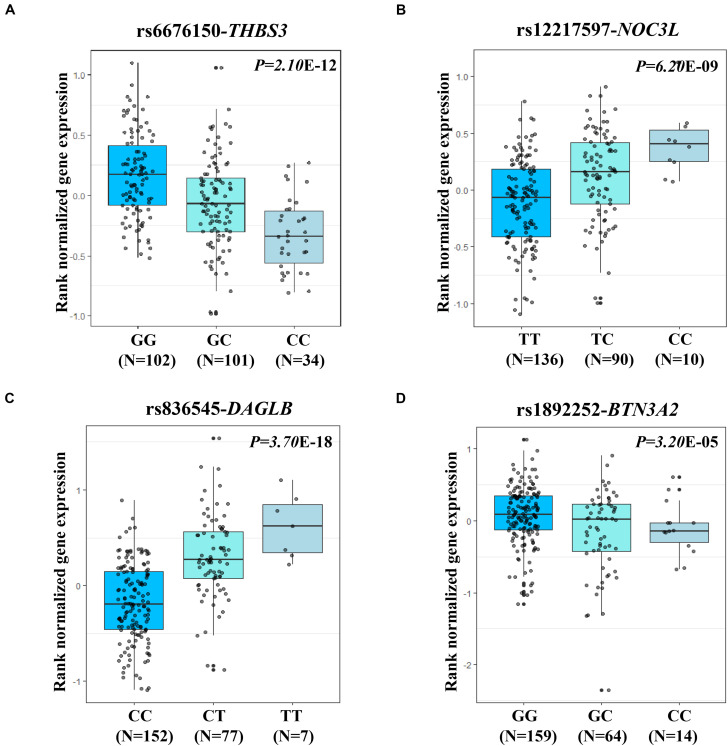
eQTL analysis shown the associations of four expression-related SNPs (eSNPs) and its related genes in stomach tissues from GTEx. The small gray dot represents the individual log2 gene expression value. **(A)** eQTL analysis (rs6676150, risk allele C) for the expression of *THBS3* (*P* = 2.10 × 10^–12^); **(B)** eQTL analysis (rs12217597, risk allele C) for the expression of *NOC3L* (*P* = 6.20 × 10^–9^); **(C)** eQTL analysis (rs836545, risk allele T) for the expression of *DAGLB* (*P* = 3.70 × 10^–18^); and **(D)** eQTL analysis (rs1892252, risk allele G) for the expression of *BTN3A2* (*P* = 3.20 × 10^–5^).

The two most strongly risk-associated variants (rs6676150 at 1q22 and rs12217597 at 10q23.33) in known loci achieved genome-wide association significance (*P* = 4.29 × 10^–10^ and *P* = 1.74 × 10^–8^, respectively), which correlated with the expression level of *THBS3* and *NOC3L*, respectively, (Figures 3A,B). Moreover, these two variants were in strong LD with previously reported index SNPs (Supplementary Table S4). Of note, we found that two novel variants at 7p22.1 (rs836545), and 6p22.2 (rs1892252) were significantly associated with GC risk (per *T* allele OR = 1.23, 95% CI: 1.12–1.35, and *P* = 7.46 × 10^–6^; per *G* allele OR = 1.41, 95% CI: 1.20–1.66, and *P* = 2.43 × 10^–5^, respectively). Meanwhile, the risk alleles rs836545-T and rs1892252-G were correlated with higher expression levels of *DAGLB* (*P* = 3.70 × 10^–18^) and *BTN3A2* (*P* = 3.20 × 10^–5^), respectively (Figures 3C,D). A total of 63 candidate SNPs in strong LD (*r*^2^ ≥ 0.6) with rs836545 were extracted by using the HaploReg v4.2 tool (Supplementary Table S5). We found that the rs836545 site located within an active enhancer in three cell types, and the variant allele was predicted to alter the binding of four regulatory motifs; however, the chromatin status in stomach tissue was quiescent. As depicted in Supplementary Figure S2, we focused on the region nearby the promoter of *DAGLB* containing two variants in perfect LD (rs3828944 and rs4724806 at a 25 bp distance, pairwise *r*^2^ = 1.00), where histone markers and chromatin state signatures exhibited a strong transcriptional activity as well as DNase-seq evidence for transcription factor binding. Using a combination of annotation tools, we proposed that rs3828944 might be the most promising functional variant in this region. We did not observe any variants in LD with the rs1892252 by HaploReg. Nevertheless, our previous study have observed a tumor-promoting role of *BTN3A2* that was remotely regulated by rs1679709 at 6p22.1 (Zhu et al., 2017).

### Variance Explained by Independent eSNPs

Based on the eSNPs identified in present study and those reported by previous GWAS, we estimated the proportion of phenotypic variance explained by a liability threshold model assuming a GC prevalence of 32.43/100,000, 42.43/100,000, and 52.43/100,000 (Table 2). These four identified eSNPs showed 0.58, 0.60, and 0.62%, respectively, while nineteen of these GWAS-reported SNPs accounted for 1.14, 1.19, and 1.23% of the total phenotypic variance at the respective prevalence. In total, all these variants associated with susceptibility to GC showed 1.30, 1.35, and 1.39% of the phenotypic variance, respectively. These two novel eSNPs (rs836545 and rs1892252) showed approximately 12.37% (0.49%/3.96%) of the phenotypic variance owing to known genetic variations.

**TABLE 2:** Heritability estimated from variants associated with GC risk.

**Model^a^**	***h*^2^(SE) observed scale**	***h*^2^(SE) liability scale**		
		**Prevalence**	**Prevalence**	**Prevalence**
		**(32.43/100,000)**	**(42.43/100,000)**	**(52.43/100,000)**
SNPs identified by previous GWAS (*n* = 19)^a^	3.50%	1.14%	1.19%	1.23%
The identified eSNPs (*n* = 4)^b^	1.80%	0.58%	0.60%	0.62%
The identified eSNPs in unknown loci (*n* = 2)^c^	0.49%	0.16%	0.16%	0.17%
Combination (*n* = 21)^d^	3.96%	1.30%	1.35%	1.39%

*^*a*^Variants reported by previous GWAS studies. ^*b*^Significant eSNPs identified by the present study. ^*c*^Significant novel eSNPs identified by the present study. ^*d*^Consist of 19 GWAS reported SNPs and 2 novel identified eSNPs by present study.*

### Susceptibility Genes Associated With GC Risk and Pathway Analysis

At the gene level, 302 (5.97%) of 5,055 pathway genes were associated with GC risk at a nominal *P*-value < 0.05. Five protein-coding genes, including *THBS3* (*P* = 2.65 × 10^–8^), *GBA* (*P* = 1.29 × 10^–6^), *GPR27* (*P* = 1.59 × 10^–5^), *AMDHD1* (*P* = 2.65 × 10^–5^), and *FBXO43* (*P* = 1.26 × 10^–4^), were significantly related to GC susceptibility in the pooled dataset after correction for multiple testing (FDR < 0.05; Table 3). Two genes (*THBS3* and *GBA*) were located in known susceptibility locus (1q22), while the other three genes (*GPR27* at 3p13, *AMDHD1* at 12q23.1, and *FBXO43* at 8q22.2) were identified as novel GC susceptibility genes. At the pathway level, there were no significant pathways after multiple testing correction. However, 23 pathways reached a less stringent threshold (*P* < 0.05), which was predominantly related to metabolism and transcription. Details are shown in Supplementary Table S6.

**TABLE 3:** Significant GC-associated protein-coding genes predicted by sequence kernel association combined test (SKAT-C).

**Region**	**Gene**	**eSNP tested^a^**	***P_*NJ*__–GWAS_***	***P_*B*__*J*__–GWAS_***	***P*_*SX–GWAS*_**	***P*_*combined*_**	**FDR^b^**
**Known region**							
1q22	*THBS3*	79	4.98 × 10^–2^	2.75 × 10^–1^	7.89 × 10^–6^	2.65 × 10^–8^	3.00 × 10^–5^
1q22	*GBA*	14	2.56 × 10^–2^	7.14 × 10^–2^	8.56 × 10^–4^	1.29 × 10^–6^	1.11 × 10^–3^
**Unknown region**							
3p13	*GPR27*	51	4.97 × 10^–8^	9.99 × 10^–1^	6.03 × 10^–3^	1.59 × 10^–5^	9.30 × 10^–3^
12q23.1	*AMDHD1*	75	1.17 × 10^–6^	2.83 × 10^–3^	1.54 × 10^–1^	2.65 × 10^–5^	1.36 × 10^–2^
8q22.2	*FBXO43*	28	3.81 × 10^–1^	5.00 × 10^–3^	1.61 × 10^–3^	1.26 × 10^–4^	4.97 × 10^–2^

*^*a*^Number of eSNPs mapped to each gene. ^*b*^False discovery rate in the combined dataset.*

### Sherlock Integrative Analysis Prioritizes Seven Risk Protein-Coding Genes

We integrated genetic associations from the meta-analysis of three GC GWAS (a total of 307,676 eSNPs with no heterogeneity) with stomach eQTL from the GTEx database. Sherlock integrative analysis identified seven top GC susceptibility genes whose expression might confer GC risk (*P* < 1.0 × 10^–3^; Table 4). Compared with the abovementioned results, this new approach validated five genes consisting of three known genes (*THBS3*, *NOC3L*, and *GBA*) and two novel genes (*FBXO43* and *DAGLB*).

**TABLE 4:** Top GC-related protein-coding genes predicted by Sherlock integrative analysis.

**Region**	**Gene**	**LBF^a^**	***P*^b^**	**Supporting SNP^c^**	***P*_*GWAS*_^d^**	***P*_*eQTL*_^e^**
**Known region**						
1q22	*THBS3*	7.31	2.45 × 10^–5^	rs2049805	2.82 × 10^–8^	1.85 × 10^–9^
10q23.33	*NOC3L*	7.18	2.45 × 10^–5^	rs12220125	2.09 × 10^–9^	2.79 × 10^–9^
1q22	*GBA*	6.87	3.43 × 10^–5^	rs12034326	1.38 × 10^–5^	2.90 × 10^–6^
**Unknown region**						
8q22.2	*FBXO43*	5.79	9.31 × 10^–5^	rs2453641	9.39 × 10^–5^	3.45 × 10^–6^
7p22.1	*DAGLB*	5.60	1.32 × 10^–4^	rs4724806	1.08 × 10^–5^	3.44 × 10^–18^
19p13.11	*HAPLN4*	4.18	7.99 × 10^–4^	rs2905421	4.48 × 10^–5^	4.62 × 10^–8^
19q13.43	*ZNF329*	4.17	8.08 × 10^–4^	rs157375	3.34 × 10^–4^	4.53 × 10^–6^

*^*a*^LBF (logarithm of Bayes factor) is to assess whether a gene is associated with GC through integrating the GWAS signal and eQTL. The larger LBF score represents the higher probability that the gene is associated with GC. For example, a LBF of 7.31 means that a gene is more likely [1495 times, (exp(7.31) = 1495] to be associated with GC than no association. ^*b*^*P*-value from Sherlock integrative analysis. ^*c*^eSNP with the highest LBF. ^*d*^*P*-value from expression quantitative trait analysis. ^*e*^*P*-value from meta-analysis of three GC GWAS datasets.*

### Differential Expression Analysis and GSEA

We compared the expression level of *DAGLB* and *FBXO43* in 32 paired tissue samples of patients with GC. Both mRNA levels of the two genes were remarkably unregulated in tumors than in their adjacent normal tissues (*P* = 5.59 × 10^–7^ and *P* = 3.90 × 10^–6^, respectively; Supplementary Figures S3A,B). The Kaplan-Meier plotter online tool revealed that high expression level of *DAGLB* or *FBXO43* was associated with an unfavorable prognosis in patients with GC (*DAGLB*, HR = 1.77, 95%CI: 1.43–2.20, and *P* = 1.30 × 10^–7^; *FBXO43*, HR = 1.39, 95%CI: 1.09–1.78, and *P* = 7.60 × 10^–3^; Supplementary Figures S3C,D). To identify the potential function of these two genes in GC tumorigenesis, we conducted GSEA on the correlation coefficients from co-expression analysis with 23,424 genes in 237 normal stomach tissues. We observed that co-expression genes with *DAGLB* or *FBXO43* were significantly enriched in several classical cancer-related pathways, including MAPK, WNT, JAK-STAT, and P53 signaling (all FDR < 0.05; Supplementary Tables S7, S8).

## Discussion

In the current study, we conducted a genome-wide scan with 2,631 cases and 4,373 controls to systematically explore the associations of 314,203 *cis*-eSNPs with GC risk, and then we incorporated the association signals with eQTL data to identify more risk genes for GC. Hitherto, this is the most extensive overview of the role of eQTL related variants in GC susceptibility. Of interest, we discovered two independent novel eSNPs associated with GC risk, which together captured nearly 12.37% of the phenotypic variance explained by all identified genetic loci. Synthesizing the results of single SNP association and gene-based analyses, we identified *DAGLB* and *FBXO43* as novel susceptibility genes for GC. Differential expression analysis and GSEA also highlighted the tumorigenicity of *DAGLB* and *FBXO43*.

At the individual eSNP level, we discovered two novel risk loci (rs836545 at 7p22.1 and rs1892252 at 6p22.2). The risk T allele of rs836545 increased the expression level of *DAGLB* in stomach tissues. As supporting evidence, it was shown that *DAGLB* was significantly elevated in GC tissues than in adjacent normal tissues. Moreover, Sherlock integrative analysis also confirmed that *DAGLB* was a promising susceptibility gene for GC. *DAGLB*, which encodes diacylglycerol lipase beta, has been widely studied in lipid mechanism. In *DAGLB* knockout mice, *DAGLβ* inhibition can reduce 2-arachidonoylglycerol and arachidonic acid and eicosanoids in macrophages (Hsu et al., 2012). A recent GWAS reported a novel variant with HDL-C levels by modifying expression of *DAGLB* (Zhou et al., 2018). To the best of our knowledge, metabolism of lipids, especially arachidonic acid, has been proved to be an important regulator in the process of inflammation and cancer (Walduck et al., 2009). Using *In silico* analysis, we identified that rs3828944 (in perfect LD with rs836545, *r*^2^ = 0.97) located in the promoter region of *DAGLB* was mapped with the center of DHS peaks in 125 cell types and within regions harboring histone marks (H3K4me1, H3K4me3, and H3K27ac) in stomach tissues or mucosae. These convergent lines of evidence implied that the risk T allele of rs3828944 at 7p22.1 might confer GC risk though enhancing the expression of *DAGLB*. For rs1892252 at 6p22.2, the risk allele rs1892252-G showed increased expression of *BTN3A2*, which was greatly overexpressed in GC tissues. A recent GWAS have reported that rs1892252-C was a risk allele for schizophrenia (OR = 1.12, 95%CI: 1.09–1.15, *P* = 7.0 × 10^–13^; Ikeda et al., 2019). Intriguingly, our group has previously verified that the rs1679709 at 6p22.1 remotely regulated *BTN3A2* expression by modulating its enhancer activity and deletion of *BTN3A2* inhibited proliferation, migration, and invasion of GC cells (Zhu et al., 2017). *BTN3A2*, an isoform of BTN3 family, participates in regulating immune signal in T and natural killer cells (Messal et al., 2011). Besides, *BTN3A2* also plays an important role in activating the phosphoantigen-mediated Vγ9Vδ2 T cells toward the development of pancreatic ductal adenocarcinoma (PDAC), implicating it as a promising immunotherapeutic target for the treatment of PDAC (Benyamine et al., 2017).

As mentioned above, only one candidate susceptibility gene was found based on single eSNP analysis. Therefore, collections of multiple genetic variants, rather than individual highly significantly associated eSNPs, may account for a putative role of the novel gene in predisposition to GC. From the results of the SKAT-C and Sherlock integrative analyses, we identified another new risk gene, *FBXO43*, also known as *EMI2*, which is a member of F-box protein family that influences the state of meiosis via translational regulation (Tan et al., 2018). A previous study has shown that the mRNA level of *FBXO43* is dramatically upregulated in hepatocellular carcinoma tissues than in normal tissues, and elevated *FBXO43* expression indicates a poor prognosis in patients with hepatocellular carcinoma (Tang et al., 2008). Consistent with the observation, *FBXO43* was overexpressed in GC tissues and associated with poor prognosis in patients with GC. Co-expression genes with *FBXO43* in normal stomach tissue were predominantly involved in several important signal transduction pathways, including MAPK, TGF-beta, WNT, and P53 signaling.

In conclusion, our findings highlighted the importance of eSNPs in dissecting genetic basis of GC. We discovered two novel eSNPs, rs836545 at 7p22.1, and rs1892252 at 6p22.2, which were significantly associated with susceptibility to GC. Furthermore, we integrated eQTL data with GWAS association signal to identify *FBXO43* and *DAGLB* as new GC risk genes. These susceptible eSNPs, together with candidate genes, will provide new insight into the genetic and biological basis for the mechanism of GC development.

## Data Availability Statement

eSNPs were derived based on stomach tissues from the GTEx database (V7 release; https://gtexportal.org/home/datasets).

## Ethics Statement

The studies involving human participants were reviewed and approved by Ethics committee of Nanjing Medical College. The patients/participants provided their written informed consent to participate in this study.

## Author Contributions

GJ and MZ designed and performed the research. CY and JN prepared the tables and figure. JN wrote the manuscript. TW and YW analyzed the data. YL, YD, BD, and GL collected the samples and information. All authors contributed to the article and approved the submitted version.

## Conflict of Interest

The authors declare that the research was conducted in the absence of any commercial or financial relationships that could be construed as a potential conflict of interest.

## Footnotes

1 https://gco.iarc.fr/today/online
