# Supplementary material for: Integration of GWAS and eQTL Analysis to Identify Risk Loci and Susceptibility Genes for Gastric Cancer
Source: Front Genet. 2020 Jul 10;11:679. doi: 10.3389/fgene.2020.00679 (PMC7366424; doi:10.3389/fgene.2020.00679)
Supplement: Supplementary file 1 [file Data_Sheet_1.docx]

**Supplementary Materials**

**Title: Integration of GWAS and Stomach eQTL Analysis to Identify Risk Loci and Target Genes for Gastric Cancer**

**Supplementary Text**

**The URLs for the data presented herein are as follows:**

SHAPEIT v2: http://mathgen.stats.ox.ac.uk/genetics_software/shapeit/shapeit.html

IMPUTE2: https://mathgen.stats.ox.ac.uk/impute/impute_v2.html

PLINK1.9: <http://www.cog-genomics.org/plink2/>

R language: <https://www.r-project.org/>

GWAMA: https://www.geenivaramu.ee/en/tools/gwama

CADD: https://cadd.gs.washington.edu/

RegulomeDB: <http://www.regulomedb.org>

PINES: <http://genetics.bwh.harvard.edu/pines/index.html>

MSigDB: [http://software.broadinstitute.org/gsea/msigdb](http://software.broadinstitute.org/gsea/msigdb/index.jsp)

Sherlock integrative analysis: <http://sherlock.ucsf.edu/>

LocusZoom: <http://locuszoom.sph.umich.edu>

Kaplan-Meier Plotter: http://www.kmplot.com/

**Supplementary Table S1.** The characteristics of participates included in three GWAS datasets.

| **Variables** | **NJ-GWAS** | |  | **BJ-GWAS** | |  | **SX-GWAS** | |
| --- | --- | --- | --- | --- | --- | --- | --- | --- |
|  | **Case (N=550)** | **Control (N=1155)** |  | **Case (N=456)** | **Control (N=1118)** |  | **Case (N=1625)** | **Control (N=2100)** |
| Age (%) |  |  |  |  |  |  |  |  |
| <60 | 292(53.09) | 583(50.48) |  | 256(56.14) | 437(39.09) |  | 731(44.98) | 1000(47.62) |
| ≥60 | 258(46.91) | 572(49.52) |  | 200(43.86) | 671(60.91) |  | 894(55.02) | 1100(52.38) |
| Sex (%) |  |  |  |  |  |  |  |  |
| Male | 392(71.27) | 823(71.26) |  | 322(70.61) | 873(78.09) |  | 1260(77.54) | 1430(68.10) |
| Female | 158(28.73) | 332(28.74) |  | 134(29.39) | 245(21.91) |  | 365(22.46) | 670(31.90) |
| Smoking (%) ^a^ |  |  |  |  |  |  |  |  |
| Yes | 250(45.45) | 565(48.92) |  | 129(28.29) | 590(52.77) |  | - | - |
| No | 300(54.55) | 590(51.08) |  | 327(71.71) | 528(47.23) |  | - | - |
| Drinking (%) ^b^ | | | | | | | | |
| Yes | 212(38.55) | 621(53.77) |  | 86(18.86) | 415(37.12) |  | - | - |
| No | 338(61.45) | 534(46.23) |  | 370(81.14) | 703(62.88) |  | - | - |

^a^ Smoking was defined as individuals who smoked at least one cigarette per day for more than one year during their lifetime, otherwise was no smoking.

^b^ Drinking was defined as individuals who drank an average of twice or more in a week for at least one year in their lifetime, otherwise was no drinking.

**Supplementary Table S2.** Summary of the gastric cancer risk-related loci reported by previous studies.

| **CHR** | **Variant*** | **Reported genes** | **Alleles** | **EAF in 1000 Genomes** | | | | **PMID** |
| --- | --- | --- | --- | --- | --- | --- | --- | --- |
|  |  |  | **(Ref>Eff)** | **AFR** | **AMR** | **ASN** | **EUR** |  |
| 1p35.2 | rs112754928 | *SPOCD1* | G>A | 0 | 0 | 0.02 | 0 | 28246015 |
| 1q22 | **rs760077** | *MUC1* | T>A | 0.36 | 0.3 | 0.17 | 0.37 | 26098866 |
|  | **rs140081212** | *MUC1* | G>A | 0.35 | 0.3 | 0.18 | 0.37 | 26098866 |
|  | **rs4072037** | *MUC1* | A>G | 0.34 | 0.34 | 0.16 | 0.41 | 20729852; 26129866; 26701879 |
|  | **rs80142782** | *ASH1L* | T>C | 0 | 0 | 0.09 | 0 | 26701879 |
| 2p11.2 | chr2:86020821 | intergenic | G>T | - | - | - | - | 26098866 |
| 3q13.31 | **rs9841504** | *ZBTB20* | C>G | 0.34 | 0.17 | 0.16 | 0.08 | 22037551 |
| 5p13.1 | **rs10074991** | *PRKAA1* | A>G | 0.67 | 0.7 | 0.46 | 0.71 | 26129866 |
|  | **rs10036575** | *PRKAA1* | C>T | 0.71 | 0.74 | 0.46 | 0.77 | 26098866 |
|  | **rs13361707** | *PRKAA1* | T>C | 0.67 | 0.7 | 0.46 | 0.71 | 22037551;26701879 |
| 5q14.3 | **rs7712641** | *lnc-POLR3G-4* | T>C | 0.32 | 0.59 | 0.58 | 0.65 | 26701879 |
| 6p21.1 | **rs2294693** | *UNC5CL, TSPO2* | T>C | 0.29 | 0.26 | 0.26 | 0.18 | 26129866 |
| 6p22.1 | **rs1679709** | *BTN3A2* | G>A | 0.23 | 0.12 | 0.14 | 0.12 | 28246015 |
| 8q24.3 | **rs2294008** | *PSCA* | C>T | 0.38 | 0.57 | 0.38 | 0.44 | 18488030;26098866;26701879 |
| 9q34.2 | **rs7849280** | *ABO* | A>G | 0.2 | 0.05 | 0.16 | 0.06 | 30281874 |
| 10q23.33 | **rs2274223** | *PLCE1, NOC3L* | A>G | 0.35 | 0.22 | 0.19 | 0.32 | 20729852 |
|  | **rs3765524** | *PLCE1, NOC3L* | C>T | 0.42 | 0.22 | 0.19 | 0.29 | 20729852 |
|  | **rs3781264** | *PLCE1, NOC3L* | T>C | 0.17 | 0.2 | 0.14 | 0.3 | 20729852 |
|  | **rs11187842** | *PLCE1, NOC3L* | C>T | 0.03 | 0.07 | 0.13 | 0.09 | 20729852 |
| 11q22.3 | chr11:108137985 | *ATM* | C>T | - | - | - | - | 26098866 |
|  | chr11:108124573 | *ATM* | C>A | - | - | - | - | 26098866 |
| 11q22.3 | chr11:102612948 | intergenic | !A>A | - | - | - | - | 26098866 |
| 12q24.11-12 | **rs6490061** | *CUX2* | C>T | 0.53 | 0.63 | 0.42 | 0.79 | 30281874 |
| 20q11.21 | **rs2376549** | *DEFB* families | T>C | 0.91 | 0.47 | 0.28 | 0.47 | 30281874 |
| 20q12 | rs55864139 | *CHD6* | T>A | 0 | 0.01 | 0 | 0.01 | 26098866 |

*Variants available in our datasets were marked in bold.

**Supplementary Table S3.** The scoring scheme refers to the following available data types for a single coordinate.

| **Score** | **Supporting data** |
| --- | --- |
| 1a | eQTL + TF binding + matched TF motif + matched DNase Footprint + DNase peak |
| 1b | eQTL + TF binding + any motif + DNase Footprint + DNase peak |
| 1c | eQTL + TF binding + matched TF motif + DNase peak |
| 1d | eQTL + TF binding + any motif + DNase peak |
| 1e | eQTL + TF binding + matched TF motif |
| 1f | eQTL + TF binding / DNase peak |
| 2a | TF binding + matched TF motif + matched DNase Footprint + DNase peak |
| 2b | TF binding + any motif + DNase Footprint + DNase peak |
| 2c | TF binding + matched TF motif + DNase peak |
| 3a | TF binding + any motif + DNase peak |
| 3b | TF binding + matched TF motif |
| 4 | TF binding + DNase peak |
| 5 | TF binding or DNase peak |
| 6 | other |

*Derived from the RegulomeDB (<http://www.regulomedb.org>).

**Supplementary Table S4.** The linkage disequilibrium (LD) between expression-related SNPs (eSNPs) in known region and GWAS reported SNPs.

| **Index eSNP** | **GWAS SNP** | **Asian^*^** | |  | **European^*^** | |
| --- | --- | --- | --- | --- | --- | --- |
|  |  | **r^2^** | **D'** |  | **r^2^** | **D'** |
| **rs6676150** | rs760077 | 0.82 | 1.00 |  | 0.89 | 0.95 |
|  | rs140081212 | 0.75 | 1.00 |  | 0.86 | 0.95 |
|  | rs4072037 | 0.79 | 1.00 |  | 0.57 | 0.91 |
|  | rs80142782 | 0.57 | 0.94 |  | - | - |
| **rs12217597** | rs2274223 | 0.55 | 0.87 |  | 0.58 | 0.96 |
|  | rs3765524 | 0.55 | 0.87 |  | 0.71 | 0.91 |
|  | rs3781264 | 0.81 | 0.96 |  | 0.71 | 0.91 |
|  | rs11187842 | 0.77 | 0.95 |  | 0.27 | 0.91 |

| *LD was calculated based on the 1000 Genome Phase 3 dataset. |
| --- |

**Supplementary Table S5.** Functional prediction of 63 variants in strong LD (r^2^≥0.6) with the lead variants rs836545 at 7p22.1.

| **RsID** | **Position**  **(hg19)** | **HaploReg v4.2** | | | | | | | **RegDB^b^** | **GWAVA** | **CADD** | **PINES** |
| --- | --- | --- | --- | --- | --- | --- | --- | --- | --- | --- | --- | --- |
|  |  | **R^2 a^** | **Alt** | **ASN** | **Promoter histone marks** | **Enhancer histone marks** | **DNAse** | **Motifs changed** |  |  |  |  |
| rs4724801 | 6412113 | 0.68 | A | 0.22 |  | GM12878 |  | LUN-1 | 6 | 0.36 | 4.12 | 2.48E-02 |
| rs33999234 | 6412387 | 0.68 | G | 0.22 |  | GM12878, K562 |  | 5 altered motifs | 7 | 0.63 | 1.69 | 4.81E-03 |
| rs34106847 | 6414732 | 0.68 | G | 0.22 | 9 cell types |  | GM12878 |  | 4 | 0.59 | 7.40 | 1.00E-04 |
| rs144179322 | 6414761 | 0.62 | TC | 0.21 | 9 cell types |  | GM12878 | 10 altered motifs | 2b | NA | 3.61 | 1.00E-04 |
| rs71531381 | 6416360 | 0.66 | C | 0.22 | 8 cell types |  |  | CACD,GR | 6 | 0.33 | 0.27 | 7.20E-04 |
| rs7456834 | 6416588 | 0.68 | C | 0.22 | 6 cell types | Huvec, K562 | 13 cell types | MAZ | 4 | 0.61 | 5.67 | 1.27E-04 |
| rs7455288 | 6416615 | 0.68 | C | 0.22 | 6 cell types | Huvec, K562 | 17 cell types | DEC,Egr-1 | 4 | 0.60 | 7.40 | 1.20E-04 |
| rs17196595 | 6418378 | 0.68 | T | 0.22 |  |  |  | 4 altered motifs | 5 | 0.20 | 0.78 | 2.50E-02 |
| rs33988121 | 6421224 | 0.67 | C | 0.22 | GM12878 | Huvec |  | 4 altered motifs | 7 | NA | 0.90 | 3.76E-02 |
| rs34884077 | 6421594 | 0.68 | G | 0.22 | GM12878 | Huvec | Th1,GM06990,  HRCEpiC |  | 4 | 0.20 | 2.98 | 3.80E-02 |
| rs11514801 | 6422248 | 0.68 | G | 0.22 |  | GM12878, Huvec |  | NRSF | 7 | 0.15 | 6.78 | 1.70E-02 |
| rs11514802 | 6422288 | 0.68 | G | 0.22 |  | Huvec, GM12878, NHEK | H7-hESC | BDP1,Nr2f2 | 5 | 0.16 | 0.76 | 1.81E-02 |
| rs10951982 | 6422556 | 0.68 | A | 0.22 |  | Huvec, NHEK, GM12878 | 12 cell types | 10 altered motifs | 4 | 0.27 | 3.14 | 5.87E-03 |
| rs2347339 | 6423724 | 0.68 | G | 0.22 | GM12878 | Huvec, NHEK |  | PLZF,RFX5 | 5 | 0.28 | 2.99 | 1.21E-02 |
| rs11514804 | 6424289 | 0.69 | G | 0.22 |  | 5 cell types |  |  | 5 | 0.25 | 8.11 | 6.97E-03 |
| rs35733871 | 6424547 | 0.68 | C | 0.22 |  | 5 cell types |  | 5 altered motifs | 7 | 0.30 | 8.46 | 6.91E-03 |
| rs34414425 | 6426318 | 0.67 | A | 0.22 |  | NHEK |  | Maf,NF-E2 | 7 | 0.28 | 6.84 | 2.63E-02 |
| rs3729790 | 6426954 | 0.68 | A | 0.22 |  | NHEK |  | Irf | 5 | 0.21 | 0.71 | 4.85E-02 |
| rs11763354 | 6428798 | 0.68 | T | 0.22 |  |  |  | HNF4,Zbtb3 | 6 | 0.07 | 2.30 | 2.10E-01 |
| rs33986648 | 6430099 | 0.68 | T | 0.22 |  |  |  | 5 altered motifs | 6 | 0.12 | 1.00 | 1.68E-01 |
| rs71531384 | 6430242 | 0.67 | A | 0.21 |  |  |  | 22 altered motifs | 6 | 0.15 | 1.48 | 1.68E-01 |
| rs1880118 | 6435220 | 0.70 | C | 0.21 |  | GM12878 |  | 7 altered motifs | 1f | 0.05 | 1.91 | 2.03E-02 |
| rs9374 | 6442371 | 0.73 | A | 0.21 |  |  |  | 6 altered motifs | 5 | 0.79 | 18.60 | NA |
| rs2303361 | 6449496 | 0.74 | C | 0.22 |  |  | Th1,PanIslets,Adult_CD4_Th0 |  | 4 | 0.48 | 6.97 | NA |
| rs35277609 | 6451041 | 0.76 | T | 0.21 |  |  |  | CEBPA,CEBPB,p300 | 6 | 0.07 | 2.73 | 5.60E-01 |
| rs35745599 | 6451080 | 0.74 | T | 0.21 |  |  |  |  | 7 | 0.07 | 1.74 | 5.66E-01 |
| rs11772942 | 6453027 | 0.78 | T | 0.22 |  |  |  | 5 altered motifs | 6 | 0.16 | 1.26 | 5.26E-01 |
| rs4724803 | 6453808 | 0.78 | G | 0.22 |  |  |  | 4 altered motifs | 6 | 0.03 | 2.08 | 5.21E-01 |
| rs4724804 | 6453845 | 0.78 | A | 0.22 |  |  |  | YY1 | 7 | 0.03 | 1.20 | 5.21E-01 |
| rs71524068 | 6455046 | 0.79 | A | 0.22 |  |  |  | Pitx2,SP2 | 6 | 0.13 | 0.64 | 4.61E-01 |
| rs836506 | 6455246 | 0.83 | G | 0.28 |  |  |  | HNF4,RXRA | 6 | 0.11 | 2.63 | 4.82E-01 |
| rs13235365 | 6456091 | 0.75 | T | 0.21 |  |  |  | 4 altered motifs | 5 | 0.52 | 0.63 | 2.35E-01 |
| rs1055428 | 6456347 | 0.70 | T | 0.20 |  |  | 17 cell types | 31 altered motifs | 2b | 0.78 | 0.01 | NA |
| rs1123675 | 6459260 | 0.80 | A | 0.22 |  | HSMM | 4 cell types | 6 altered motifs | 2b | 0.30 | 4.75 | 5.75E-02 |
| rs12532493 | 6461914 | 0.80 | A | 0.22 |  |  | 8988T,Monocytes-CD14+_RO01746 | 7 altered motifs | 4 | 0.35 | 0.78 | 9.62E-02 |
| rs12537483 | 6461965 | 0.80 | A | 0.22 |  |  | 8988T,iPS,Caco-2 | Ets,Hic1,ZNF263 | 4 | 0.36 | 2.01 | 9.24E-02 |
| rs113080138 | 6463365 | 0.78 | G | 0.22 |  |  |  | CEBPB,Rad21 | 7 | 0.08 | 0.84 | 7.07E-02 |
| rs12539027 | 6464147 | 0.79 | C | 0.22 |  | GM12878, NHEK | 9 cell types | 16 altered motifs | 2b | 0.52 | 0.02 | 6.35E-03 |
| rs11767078 | 6464788 | 0.80 | A | 0.22 |  |  | HeLa-S3,Th1,Caco-2 | AIRE,Pax-5 | 5 | 0.27 | 0.05 | 1.79E-02 |
| rs11760556 | 6464836 | 0.80 | T | 0.22 |  |  | Th1,Caco-2 | BCL,HEY1,Nanog | 5 | 0.32 | 3.59 | 3.26E-02 |
| rs11760562 | 6464883 | 0.80 | T | 0.22 |  |  | 4 cell types | GR,Pax-4,Zbtb3 | 5 | 0.28 | 1.89 | 2.98E-02 |
| rs2272157 | 6465441 | 0.80 | T | 0.22 |  |  | LNCaP,GM19240 | CTCF,PPAR | 5 | 0.21 | 2.99 | 7.67E-02 |
| rs71531392 | 6466636 | 0.80 | T | 0.22 |  | GM12878 |  | Maf | 6 | 0.02 | 0.72 | 4.26E-01 |
| rs12537894 | 6467451 | 0.78 | T | 0.22 |  |  | Th1,FibroP | Nkx3,Zfp691 | 5 | 0.06 | 0.09 | 4.70E-01 |
| rs35444732 | 6473964 | 0.80 | C | 0.22 |  |  | Fibrobl,Monocytes-CD14+_RO01746 |  | 5 | 0.29 | 1.08 | 3.03E-02 |
| rs12538142 | 6475799 | 0.80 | C | 0.22 |  | Huvec, GM12878 |  | Pax-2,Pou3f3 | 4 | 0.47 | 4.63 | 4.96E-03 |
| rs3750038 | 6476237 | 0.80 | T | 0.22 |  | GM12878 | HMVEC-LBl | Foxp3,Pou2f2,TEF | 3a | 0.53 | 1.93 | 4.59E-03 |
| rs58072643 | 6476500 | 0.80 | A | 0.22 |  | GM12878 |  | 8 altered motifs | 6 | 0.36 | 0.35 | 1.41E-02 |
| rs192525127 | 6476801 | 0.65 | A | 0.22 |  |  |  | 16 altered motifs | 6 | 0.15 | 2.61 | 2.54E-02 |
| rs12535659 | 6476915 | 0.78 | T | 0.23 |  | NHEK | 4 cell types | Foxo,Myc,Pou2f2 | 5 | 0.35 | 0.83 | 1.49E-02 |
| rs13221129 | 6477136 | 0.78 | T | 0.22 |  | HepG2 | 10 cell types | 18 altered motifs | 2c | 0.28 | 2.64 | 1.84E-02 |
| rs66796060 | 6478491 | 0.81 | G | 0.22 |  | 4 cell types |  | 5 altered motifs | 5 | NA | 0.86 | 2.26E-02 |
| rs836545 | 6479410 | 1.00 | C | 0.26 |  | HSMM, NHLF, Huvec |  | 4 altered motifs | 7 | 0.15 | 0.62 | 7.12E-02 |
| rs836546 | 6479668 | 0.98 | A | 0.26 |  |  |  | Ik-2,NF-AT1 | 7 | 0.13 | 0.14 | 9.90E-02 |
| rs7797644 | 6486067 | 0.97 | C | 0.74 |  |  |  | Zbtb3 | 7 | 0.33 | 2.45 | 2.66E-03 |
| **rs4724806** | 6487131 | 0.97 | C | 0.74 | 9 cell types |  | 12 cell types | NRSF | 4 | 0.80 | 2.16 | 2.00E-05 |
| **rs3828944** | 6487156 | 0.97 | C | 0.74 | 9 cell types |  | 10 cell types | 11 altered motifs | 2b | 0.70 | 4.70 | 6.67E-06 |
| rs7807755 | 6488097 | 0.96 | C | 0.74 | 7 cell types | Huvec, HMEC | T-47D,GM12864 | GLI,Ik-1 | 4 | 0.81 | 7.89 | 3.33E-05 |
| rs4724808 | 6491806 | 0.71 | T | 0.72 |  | K562 |  |  | 7 | 0.23 | 1.03 | 1.26E-01 |
| rs4720674 | 6492046 | 0.72 | T | 0.72 |  | K562 |  | BCL,Pax-5,Pbx3 | 5 | 0.08 | 6.84 | 8.43E-02 |
| rs7810553 | 6492879 | 0.72 | T | 0.72 |  |  |  | CHOP::CEBPalpha,Dobox4,Hoxb13 | 7 | 0.13 | 0.26 | 8.86E-02 |
| rs4724809 | 6493888 | 0.72 | C | 0.72 |  | 4 cell types |  | Maf,NRSF | 5 | 0.08 | 0.14 | 8.09E-02 |
| rs4724810 | 6493945 | 0.72 | C | 0.72 |  | 4 cell types |  |  | 7 | 0.04 | 0.84 | 8.34E-02 |

^a^ LD value of r^2^ with the lead variant rs836545 in Asian of the 1000 Genomes.

^b^ RegDB: RegulomeDB.

**Supplementary Table S6.** Pathway analysis in the merged dataset (*P*<0.05).

| **Database** | **Pathway** | **Numbers of Gene** | **P_value^a^** |
| --- | --- | --- | --- |
| KEGG | Other_glycan_degradation | 16 | 7.10E-03 |
| REACTOME | Glycosphingolipid_metabolism | 38 | 1.98E-02 |
| KEGG | Sphingolipid_metabolism | 40 | 2.12E-02 |
| KEGG | Histidine_metabolism | 29 | 2.35E-02 |
| BIOCARTA | Feeder_pathway | 9 | 2.53E-02 |
| BIOCARTA | Biocarta_ARF_pathway | 17 | 2.65E-02 |
| REACTOME | RNA_pol_iii_chain_elongation | 17 | 2.66E-02 |
| KEGG | TGF_beta_signaling_pathway | 86 | 2.74E-02 |
| KEGG | ECM_receptor_interaction | 84 | 3.02E-02 |
| REACTOME | RNA_pol_iii_transcription_termination | 19 | 3.03E-02 |
| REACTOME | Digestion_of_dietary_carbohydrate | 12 | 3.29E-02 |
| REACTOME | Reactome_activation_of_IRF3_IRF6_mediated_by_TBK1_IKK_epsilon | 14 | 3.31E-02 |
| REACTOME | Signaling_by_PDGF | 122 | 3.41E-02 |
| REACTOME | TRAF3_dependent_IRF_activation_pathway | 14 | 3.43E-02 |
| REACTOME | RNA_pol_i_transcription_termination | 22 | 3.46E-02 |
| REACTOME | Sphingolipid_metabolism | 69 | 3.50E-02 |
| REACTOME | RNA_pol_iii_transcription_initiation_from_type_2_promoter | 23 | 3.71E-02 |
| REACTOME | RNA_pol_i_transcription_initiation | 25 | 3.98E-02 |
| REACTOME | RNA_pol_iii_transcription_initiation_from_type_3_promoter | 26 | 4.04E-02 |
| REACTOME | Regulation_of_rheb_gtpase_activity_by_ampk | 10 | 4.46E-02 |
| REACTOME | Effects_of_pip2_hydrolysis | 25 | 4.65E-02 |
| KEGG | RNA_polymerase | 29 | 4.70E-02 |
| BIOCARTA | Leptin_pathway | 11 | 4.76E-02 |

^a^*P* values were derived from ARTP algorithms based on 10,000 permutations.

**Supplementary Table S7.** GSEA enrichment results of co-expressed genes with *DAGLB* (FDR<0.05).

| **Pathways** | **SetSize** | **EnrichmentScore** | **NES** | **P_value** | **FDR** |
| --- | --- | --- | --- | --- | --- |
| LYSOSOME | 107 | 0.45 | 2.37 | 1.96E-05 | 1.60E-04 |
| OXIDATIVE_PHOSPHORYLATION | 94 | 0.44 | 2.27 | 1.97E-05 | 1.60E-04 |
| PEROXISOME | 65 | 0.48 | 2.26 | 1.97E-05 | 1.60E-04 |
| ARRHYTHMOGENIC_RIGHT_VENTRICULAR_CARDIOMYOPATHY_ARVC | 63 | -0.47 | -2.21 | 2.03E-05 | 1.60E-04 |
| VASCULAR_SMOOTH_MUSCLE_CONTRACTION | 90 | -0.50 | -2.53 | 2.04E-05 | 1.60E-04 |
| FOCAL_ADHESION | 170 | -0.39 | -2.21 | 2.04E-05 | 1.60E-04 |
| HYPERTROPHIC_CARDIOMYOPATHY_HCM | 70 | -0.48 | -2.32 | 2.04E-05 | 1.60E-04 |
| NEUROACTIVE_LIGAND_RECEPTOR_INTERACTION | 166 | -0.39 | -2.23 | 2.04E-05 | 1.60E-04 |
| DILATED_CARDIOMYOPATHY | 74 | -0.51 | -2.51 | 2.04E-05 | 1.60E-04 |
| ECM_RECEPTOR_INTERACTION | 74 | -0.49 | -2.39 | 2.04E-05 | 1.60E-04 |
| **CALCIUM_SIGNALING_PATHWAY** | 138 | -0.43 | -2.37 | 2.04E-05 | 1.60E-04 |
| **MAPK_SIGNALING_PATHWAY** | 209 | -0.34 | -1.99 | 2.04E-05 | 1.60E-04 |
| PENTOSE_AND_GLUCURONATE_INTERCONVERSIONS | 15 | 0.73 | 2.33 | 3.95E-05 | 2.93E-04 |
| AMINOACYL_TRNA_BIOSYNTHESIS | 40 | 0.54 | 2.27 | 5.88E-05 | 4.08E-04 |
| N_GLYCAN_BIOSYNTHESIS | 41 | 0.50 | 2.15 | 7.83E-05 | 5.16E-04 |
| BASE_EXCISION_REPAIR | 28 | 0.57 | 2.18 | 9.82E-05 | 6.19E-04 |
| PORPHYRIN_AND_CHLOROPHYLL_METABOLISM | 25 | 0.59 | 2.21 | 9.83E-05 | 6.19E-04 |
| STEROID_HORMONE_BIOSYNTHESIS | 36 | 0.52 | 2.15 | 9.84E-05 | 6.19E-04 |
| GAP_JUNCTION | 70 | -0.41 | -1.98 | 1.02E-04 | 6.29E-04 |
| HUNTINGTONS_DISEASE | 133 | 0.34 | 1.86 | 1.18E-04 | 7.22E-04 |
| METABOLISM_OF_XENOBIOTICS_BY_CYTOCHROME_P450 | 51 | 0.46 | 2.04 | 1.37E-04 | 8.23E-04 |
| CELL_CYCLE | 106 | 0.35 | 1.84 | 2.16E-04 | 1.21E-03 |
| **TGF_BETA_SIGNALING_PATHWAY** | 74 | -0.39 | -1.91 | 2.65E-04 | 1.43E-03 |
| LONG_TERM_DEPRESSION | 50 | -0.43 | -1.95 | 3.06E-04 | 1.62E-03 |
| FRUCTOSE_AND_MANNOSE_METABOLISM | 30 | 0.53 | 2.08 | 3.33E-04 | 1.73E-03 |
| DRUG_METABOLISM_CYTOCHROME_P450 | 50 | 0.43 | 1.91 | 4.31E-04 | 2.15E-03 |
| DNA_REPLICATION | 30 | 0.50 | 1.97 | 1.00E-03 | 4.33E-03 |
| OLFACTORY_TRANSDUCTION | 35 | -0.47 | -1.92 | 1.10E-03 | 4.67E-03 |
| GLUTATHIONE_METABOLISM | 39 | 0.45 | 1.89 | 1.18E-03 | 4.91E-03 |
| FATTY_ACID_METABOLISM | 39 | 0.45 | 1.88 | 1.32E-03 | 5.34E-03 |
| PARKINSONS_DISEASE | 90 | 0.34 | 1.73 | 1.40E-03 | 5.58E-03 |
| RETINOL_METABOLISM | 42 | 0.43 | 1.86 | 1.47E-03 | 5.80E-03 |
| BUTANOATE_METABOLISM | 29 | 0.49 | 1.92 | 1.71E-03 | 6.58E-03 |
| ALZHEIMERS_DISEASE | 126 | 0.30 | 1.61 | 2.29E-03 | 8.40E-03 |
| VALINE_LEUCINE_AND_ISOLEUCINE_DEGRADATION | 40 | 0.43 | 1.81 | 2.63E-03 | 9.38E-03 |
| GLYCOSYLPHOSPHATIDYLINOSITOL_GPI_ANCHOR_BIOSYNTHESIS | 19 | 0.55 | 1.89 | 2.92E-03 | 1.02E-02 |
| DRUG_METABOLISM_OTHER_ENZYMES | 29 | 0.47 | 1.84 | 2.98E-03 | 1.04E-02 |
| SPHINGOLIPID_METABOLISM | 32 | 0.46 | 1.83 | 3.05E-03 | 1.05E-02 |
| PROTEASOME | 37 | 0.44 | 1.80 | 3.06E-03 | 1.06E-02 |
| STEROID_BIOSYNTHESIS | 14 | 0.61 | 1.91 | 3.32E-03 | 1.13E-02 |
| PRIMARY_IMMUNODEFICIENCY | 23 | 0.51 | 1.85 | 3.38E-03 | 1.15E-02 |
| **WNT_SIGNALING_PATHWAY** | 128 | -0.29 | -1.58 | 3.53E-03 | 1.18E-02 |
| OTHER_GLYCAN_DEGRADATION | 15 | 0.58 | 1.87 | 4.76E-03 | 1.49E-02 |
| AXON_GUIDANCE | 111 | -0.30 | -1.58 | 4.87E-03 | 1.52E-02 |
| SYSTEMIC_LUPUS_ERYTHEMATOSUS | 72 | -0.34 | -1.65 | 5.01E-03 | 1.55E-02 |
| CELL_ADHESION_MOLECULES_CAMS | 105 | -0.30 | -1.58 | 5.11E-03 | 1.58E-02 |
| ASCORBATE_AND_ALDARATE_METABOLISM | 13 | 0.61 | 1.85 | 5.39E-03 | 1.64E-02 |
| MELANOGENESIS | 85 | -0.32 | -1.60 | 5.71E-03 | 1.71E-02 |
| **PATHWAYS_IN_CANCER** | 275 | -0.23 | -1.39 | 8.59E-03 | 2.41E-02 |
| CITRATE_CYCLE_TCA_CYCLE | 24 | 0.47 | 1.73 | 9.41E-03 | 2.59E-02 |
| LONG_TERM_POTENTIATION | 52 | -0.36 | -1.62 | 1.00E-02 | 2.72E-02 |
| NON_HOMOLOGOUS_END_JOINING | 11 | 0.61 | 1.78 | 1.03E-02 | 2.76E-02 |
| REGULATION_OF_ACTIN_CYTOSKELETON | 171 | -0.25 | -1.44 | 1.05E-02 | 2.82E-02 |
| PYRIMIDINE_METABOLISM | 82 | 0.31 | 1.54 | 1.14E-02 | 3.00E-02 |
| PRION_DISEASES | 23 | -0.47 | -1.72 | 1.16E-02 | 3.06E-02 |
| GLYCOSPHINGOLIPID_BIOSYNTHESIS_LACTO_AND_NEOLACTO_SERIES | 22 | 0.47 | 1.67 | 1.61E-02 | 3.91E-02 |
| RNA_POLYMERASE | 25 | 0.44 | 1.64 | 1.81E-02 | 4.26E-02 |
| GLYCEROPHOSPHOLIPID_METABOLISM | 62 | 0.33 | 1.52 | 1.82E-02 | 4.27E-02 |
| RENIN_ANGIOTENSIN_SYSTEM | 12 | -0.57 | -1.69 | 2.03E-02 | 4.66E-02 |
| AMINO_SUGAR_AND_NUCLEOTIDE_SUGAR_METABOLISM | 39 | 0.37 | 1.56 | 2.17E-02 | 4.94E-02 |

**Supplementary Table S8.** GSEA enrichment results of co-expressed genes with *FBXO43* (FDR<0.05).

| **Pathway** | **SetSize** | **EnrichmentScore** | **NES** | **P_value** | **FDR** |
| --- | --- | --- | --- | --- | --- |
| **PATHWAYS_IN_CANCER** | 308 | -0.30 | -1.92 | 1.75E-05 | 1.25E-04 |
| **MAPK_SIGNALING_PATHWAY** | 242 | -0.40 | -2.45 | 1.77E-05 | 1.25E-04 |
| REGULATION_OF_ACTIN_CYTOSKELETON | 197 | -0.36 | -2.11 | 1.80E-05 | 1.25E-04 |
| FOCAL_ADHESION | 195 | -0.43 | -2.57 | 1.80E-05 | 1.25E-04 |
| **WNT_SIGNALING_PATHWAY** | 141 | -0.37 | -2.11 | 1.82E-05 | 1.25E-04 |
| NEUROTROPHIN_SIGNALING_PATHWAY | 125 | -0.40 | -2.18 | 1.82E-05 | 1.25E-04 |
| VASCULAR_SMOOTH_MUSCLE_CONTRACTION | 108 | -0.43 | -2.32 | 1.84E-05 | 1.25E-04 |
| DILATED_CARDIOMYOPATHY | 84 | -0.48 | -2.45 | 1.85E-05 | 1.25E-04 |
| **TGF_BETA_SIGNALING_PATHWAY** | 84 | -0.45 | -2.30 | 1.85E-05 | 1.25E-04 |
| ECM_RECEPTOR_INTERACTION | 80 | -0.45 | -2.27 | 1.86E-05 | 1.25E-04 |
| GAP_JUNCTION | 80 | -0.43 | -2.16 | 1.86E-05 | 1.25E-04 |
| HYPERTROPHIC_CARDIOMYOPATHY_HCM | 78 | -0.47 | -2.36 | 1.86E-05 | 1.25E-04 |
| ARRHYTHMOGENIC_RIGHT_VENTRICULAR_CARDIOMYOPATHY_ARVC | 70 | -0.45 | -2.19 | 1.86E-05 | 1.25E-04 |
| PENTOSE_AND_GLUCURONATE_INTERCONVERSIONS | 16 | 0.71 | 2.39 | 2.10E-05 | 1.25E-04 |
| BUTANOATE_METABOLISM | 30 | 0.60 | 2.43 | 2.13E-05 | 1.25E-04 |
| BASE_EXCISION_REPAIR | 33 | 0.54 | 2.26 | 2.14E-05 | 1.25E-04 |
| VALINE_LEUCINE_AND_ISOLEUCINE_DEGRADATION | 44 | 0.50 | 2.24 | 2.14E-05 | 1.25E-04 |
| DRUG_METABOLISM_CYTOCHROME_P450 | 55 | 0.52 | 2.50 | 2.14E-05 | 1.25E-04 |
| METABOLISM_OF_XENOBIOTICS_BY_CYTOCHROME_P450 | 54 | 0.53 | 2.50 | 2.14E-05 | 1.25E-04 |
| RIBOSOME | 85 | -0.39 | -2.00 | 3.70E-05 | 1.78E-04 |
| STEROID_HORMONE_BIOSYNTHESIS | 39 | 0.49 | 2.15 | 4.28E-05 | 2.00E-04 |
| PEROXISOME | 74 | 0.41 | 2.09 | 6.47E-05 | 2.79E-04 |
| PATHOGENIC_ESCHERICHIA_COLI_INFECTION | 53 | -0.44 | -2.02 | 1.13E-04 | 4.36E-04 |
| PRIMARY_IMMUNODEFICIENCY | 31 | 0.53 | 2.19 | 1.28E-04 | 4.90E-04 |
| OXIDATIVE_PHOSPHORYLATION | 111 | 0.34 | 1.86 | 1.32E-04 | 4.97E-04 |
| CALCIUM_SIGNALING_PATHWAY | 164 | -0.31 | -1.81 | 1.45E-04 | 5.42E-04 |
| SPLICEOSOME | 124 | -0.33 | -1.82 | 1.64E-04 | 6.02E-04 |
| DNA_REPLICATION | 36 | 0.48 | 2.07 | 1.71E-04 | 6.18E-04 |
| RENAL_CELL_CARCINOMA | 70 | -0.39 | -1.90 | 2.61E-04 | 8.96E-04 |
| GLUTATHIONE_METABOLISM | 46 | 0.44 | 2.01 | 2.78E-04 | 9.48E-04 |
| ADHERENS_JUNCTION | 73 | -0.38 | -1.88 | 3.35E-04 | 1.10E-03 |
| ASCORBATE_AND_ALDARATE_METABOLISM | 14 | 0.66 | 2.13 | 4.83E-04 | 1.50E-03 |
| PORPHYRIN_AND_CHLOROPHYLL_METABOLISM | 29 | 0.51 | 2.05 | 5.11E-04 | 1.58E-03 |
| RETINOL_METABOLISM | 44 | 0.44 | 1.97 | 5.99E-04 | 1.80E-03 |
| N_GLYCAN_BIOSYNTHESIS | 46 | 0.43 | 1.95 | 6.20E-04 | 1.84E-03 |
| **TOLL_LIKE_RECEPTOR_SIGNALING_PATHWAY** | 82 | -0.36 | -1.81 | 6.48E-04 | 1.91E-03 |
| FRUCTOSE_AND_MANNOSE_METABOLISM | 34 | 0.48 | 2.01 | 6.83E-04 | 2.00E-03 |
| STEROID_BIOSYNTHESIS | 17 | 0.60 | 2.07 | 7.38E-04 | 2.12E-03 |
| **CHEMOKINE_SIGNALING_PATHWAY** | 166 | -0.29 | -1.67 | 8.13E-04 | 2.28E-03 |
| TIGHT_JUNCTION | 124 | -0.31 | -1.68 | 8.76E-04 | 2.42E-03 |
| PRION_DISEASES | 30 | -0.49 | -1.97 | 9.22E-04 | 2.52E-03 |
| **JAK_STAT_SIGNALING_PATHWAY** | 118 | -0.31 | -1.69 | 9.34E-04 | 2.54E-03 |
| AMINOACYL_TRNA_BIOSYNTHESIS | 41 | 0.44 | 1.93 | 9.85E-04 | 2.66E-03 |
| CELL_CYCLE | 122 | 0.30 | 1.69 | 1.17E-03 | 3.07E-03 |
| LONG_TERM_DEPRESSION | 62 | -0.38 | -1.80 | 1.19E-03 | 3.12E-03 |
| LEUKOCYTE_TRANSENDOTHELIAL_MIGRATION | 107 | -0.31 | -1.68 | 1.34E-03 | 3.46E-03 |
| LYSOSOME | 120 | 0.30 | 1.68 | 1.37E-03 | 3.51E-03 |
| CHRONIC_MYELOID_LEUKEMIA | 72 | -0.36 | -1.77 | 1.38E-03 | 3.53E-03 |
| MATURITY_ONSET_DIABETES_OF_THE_YOUNG | 17 | 0.58 | 2.00 | 1.58E-03 | 3.94E-03 |
| DRUG_METABOLISM_OTHER_ENZYMES | 35 | 0.45 | 1.90 | 1.60E-03 | 3.98E-03 |
| FATTY_ACID_METABOLISM | 42 | 0.41 | 1.84 | 2.05E-03 | 4.93E-03 |
| MELANOGENESIS | 94 | -0.32 | -1.68 | 2.38E-03 | 5.57E-03 |
| INSULIN_SIGNALING_PATHWAY | 131 | -0.29 | -1.62 | 2.41E-03 | 5.62E-03 |
| PROSTATE_CANCER | 86 | -0.33 | -1.69 | 2.64E-03 | 6.11E-03 |
| AXON_GUIDANCE | 123 | -0.29 | -1.61 | 2.68E-03 | 6.18E-03 |
| UBIQUITIN_MEDIATED_PROTEOLYSIS | 133 | -0.29 | -1.60 | 2.91E-03 | 6.61E-03 |
| DORSO_VENTRAL_AXIS_FORMATION | 22 | -0.52 | -1.89 | 2.93E-03 | 6.66E-03 |
| BASAL_TRANSCRIPTION_FACTORS | 31 | -0.46 | -1.84 | 3.16E-03 | 7.08E-03 |
| ARACHIDONIC_ACID_METABOLISM | 50 | 0.38 | 1.77 | 3.32E-03 | 7.38E-03 |
| ERBB_SIGNALING_PATHWAY | 86 | -0.32 | -1.65 | 3.87E-03 | 8.35E-03 |
| PHOSPHATIDYLINOSITOL_SIGNALING_SYSTEM | 75 | -0.33 | -1.66 | 4.77E-03 | 9.94E-03 |
| OTHER_GLYCAN_DEGRADATION | 15 | 0.57 | 1.89 | 4.80E-03 | 1.00E-02 |
| GLYCOSYLPHOSPHATIDYLINOSITOL_GPI_ANCHOR_BIOSYNTHESIS | 23 | 0.48 | 1.81 | 5.80E-03 | 1.18E-02 |
| LINOLEIC_ACID_METABOLISM | 24 | 0.47 | 1.80 | 6.25E-03 | 1.26E-02 |
| LONG_TERM_POTENTIATION | 64 | -0.34 | -1.64 | 6.80E-03 | 1.35E-02 |
| TERPENOID_BACKBONE_BIOSYNTHESIS | 14 | 0.57 | 1.83 | 7.26E-03 | 1.42E-02 |
| HOMOLOGOUS_RECOMBINATION | 28 | 0.44 | 1.75 | 7.67E-03 | 1.48E-02 |
| SELENOAMINO_ACID_METABOLISM | 26 | 0.45 | 1.76 | 7.78E-03 | 1.49E-02 |
| PROPANOATE_METABOLISM | 31 | 0.42 | 1.73 | 7.82E-03 | 1.50E-02 |
| VASOPRESSIN_REGULATED_WATER_REABSORPTION | 42 | -0.39 | -1.68 | 8.41E-03 | 1.59E-02 |
| ACUTE_MYELOID_LEUKEMIA | 56 | -0.35 | -1.63 | 8.52E-03 | 1.61E-02 |
| **SMALL_CELL_LUNG_CANCER** | 83 | -0.30 | -1.55 | 1.06E-02 | 1.94E-02 |
| HISTIDINE_METABOLISM | 28 | 0.43 | 1.71 | 1.11E-02 | 2.01E-02 |
| **P53_SIGNALING_PATHWAY** | 68 | 0.31 | 1.56 | 1.27E-02 | 2.24E-02 |
| MISMATCH_REPAIR | 23 | 0.45 | 1.70 | 1.37E-02 | 2.38E-02 |
| LEISHMANIA_INFECTION | 67 | -0.32 | -1.55 | 1.51E-02 | 2.57E-02 |
| GLYCINE_SERINE_AND_THREONINE_METABOLISM | 29 | 0.41 | 1.66 | 1.60E-02 | 2.68E-02 |
| NEUROACTIVE_LIGAND_RECEPTOR_INTERACTION | 194 | -0.23 | -1.39 | 1.84E-02 | 3.00E-02 |
| GLYOXYLATE_AND_DICARBOXYLATE_METABOLISM | 14 | 0.52 | 1.70 | 1.93E-02 | 3.11E-02 |
| GLIOMA | 64 | -0.31 | -1.51 | 2.05E-02 | 3.25E-02 |
| COLORECTAL_CANCER | 61 | -0.32 | -1.52 | 2.12E-02 | 3.35E-02 |
| ENDOMETRIAL_CANCER | 51 | -0.34 | -1.54 | 2.19E-02 | 3.45E-02 |
| MTOR_SIGNALING_PATHWAY | 51 | -0.33 | -1.52 | 2.53E-02 | 3.89E-02 |
| SULFUR_METABOLISM | 13 | 0.51 | 1.62 | 3.09E-02 | 4.55E-02 |
| MELANOMA | 62 | -0.30 | -1.46 | 3.35E-02 | 4.85E-02 |

**
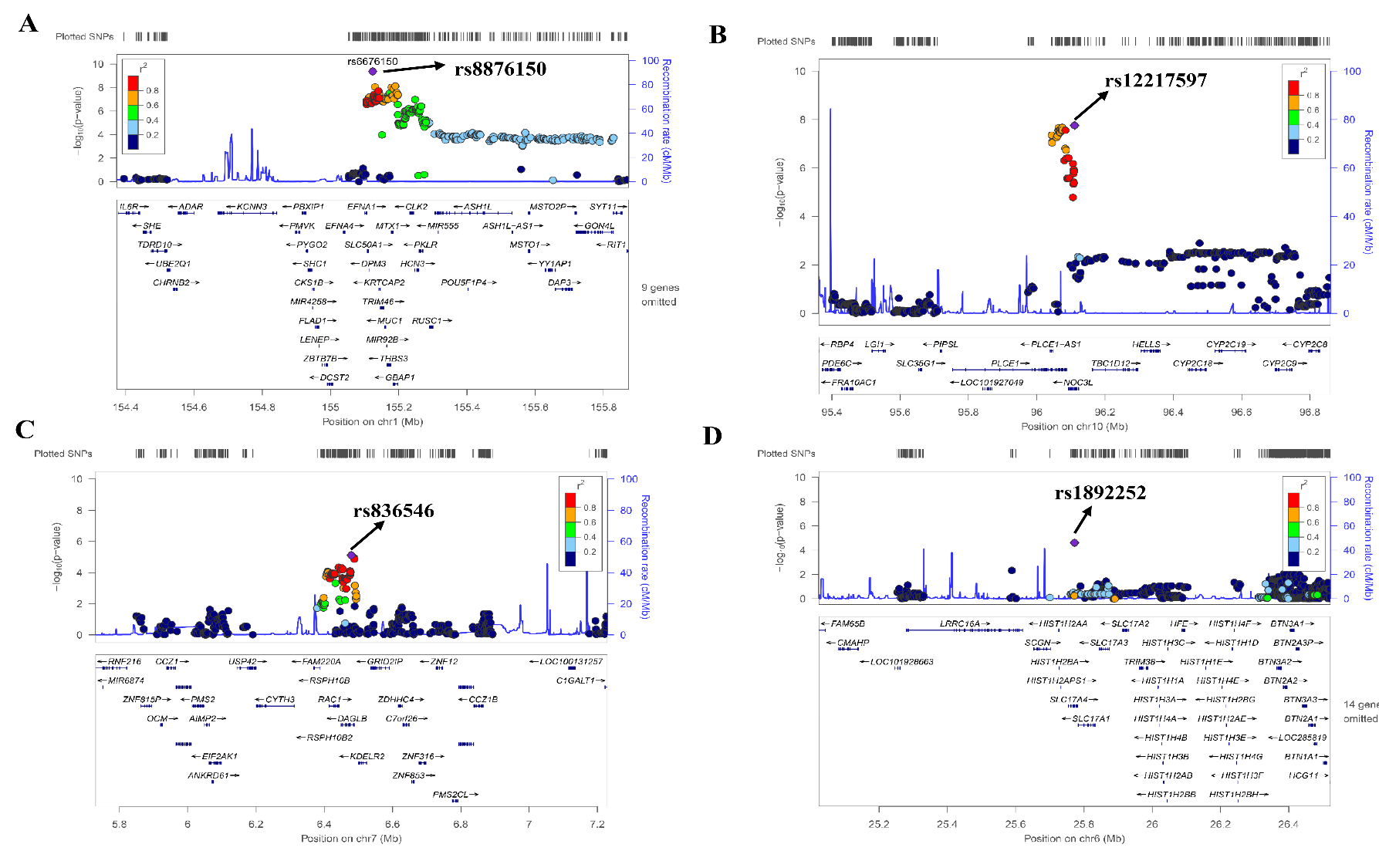
**

**Supplementary Figure S1.** Regional association plots of (A) rs6676150 at 1q22, (B) rs12217597 at 10q23.33, (C) rs836545 at 7p22.1, (D) rs1892252 at 6p22.2. Expression-related SNPs (eSNPs) are plotted according to their position with –log10 *P* values from the meta-analysis of three GWAS in the region flanking 750kb on either side of the marker SNP. The right y axis shows the recombination rate calculated from the 1000 Genome Project CHB and JPT data.

**
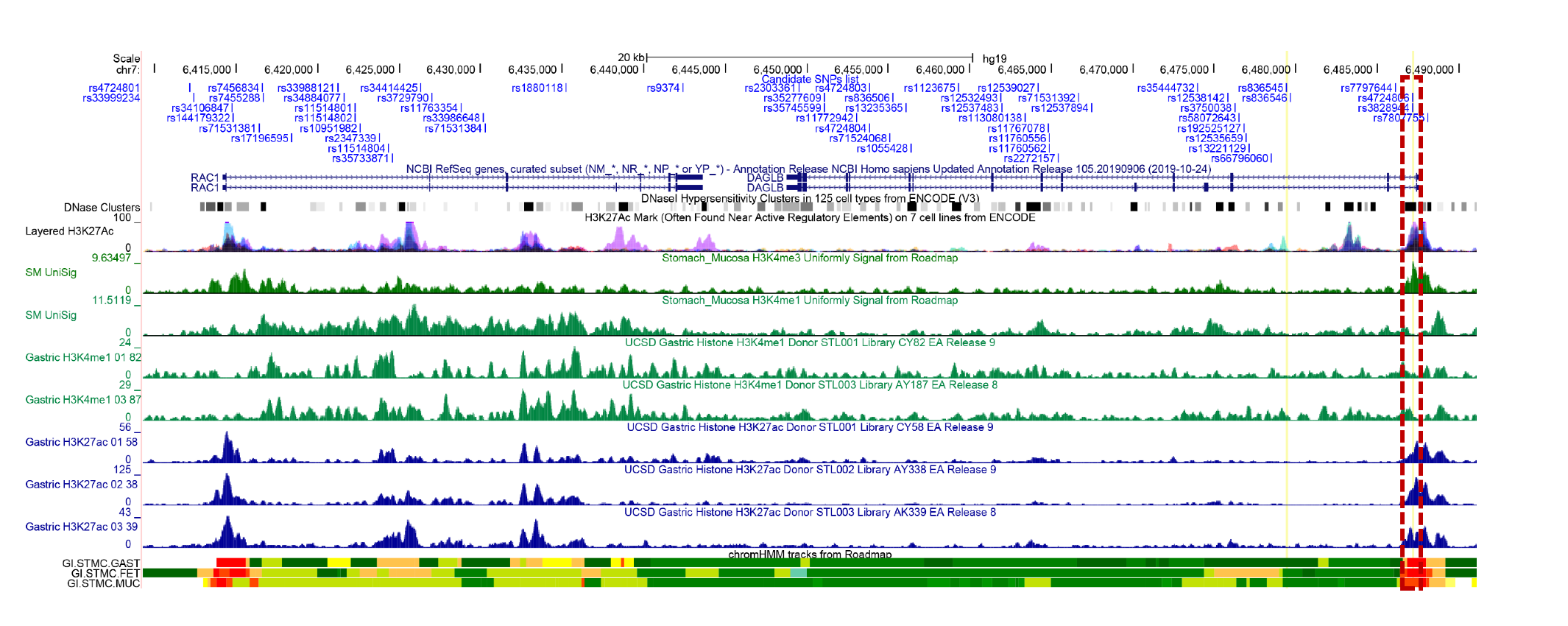
**

**Supplementary Figure S2.** Functional annotation of rs836545 at 7p22.1: ChIP-seq data in stomach tissues show enrichments of histone markers, DHSs in the site of rs3828944 which is in high LD with the lead variant rs836545 (r^2^=0.97). The chromatin state segmentation data from fetal, adult stomach tissues and mucosa tissues also confirmed this site was located in a promoter/TSS region (chromHMM tracks were in Orange Red).


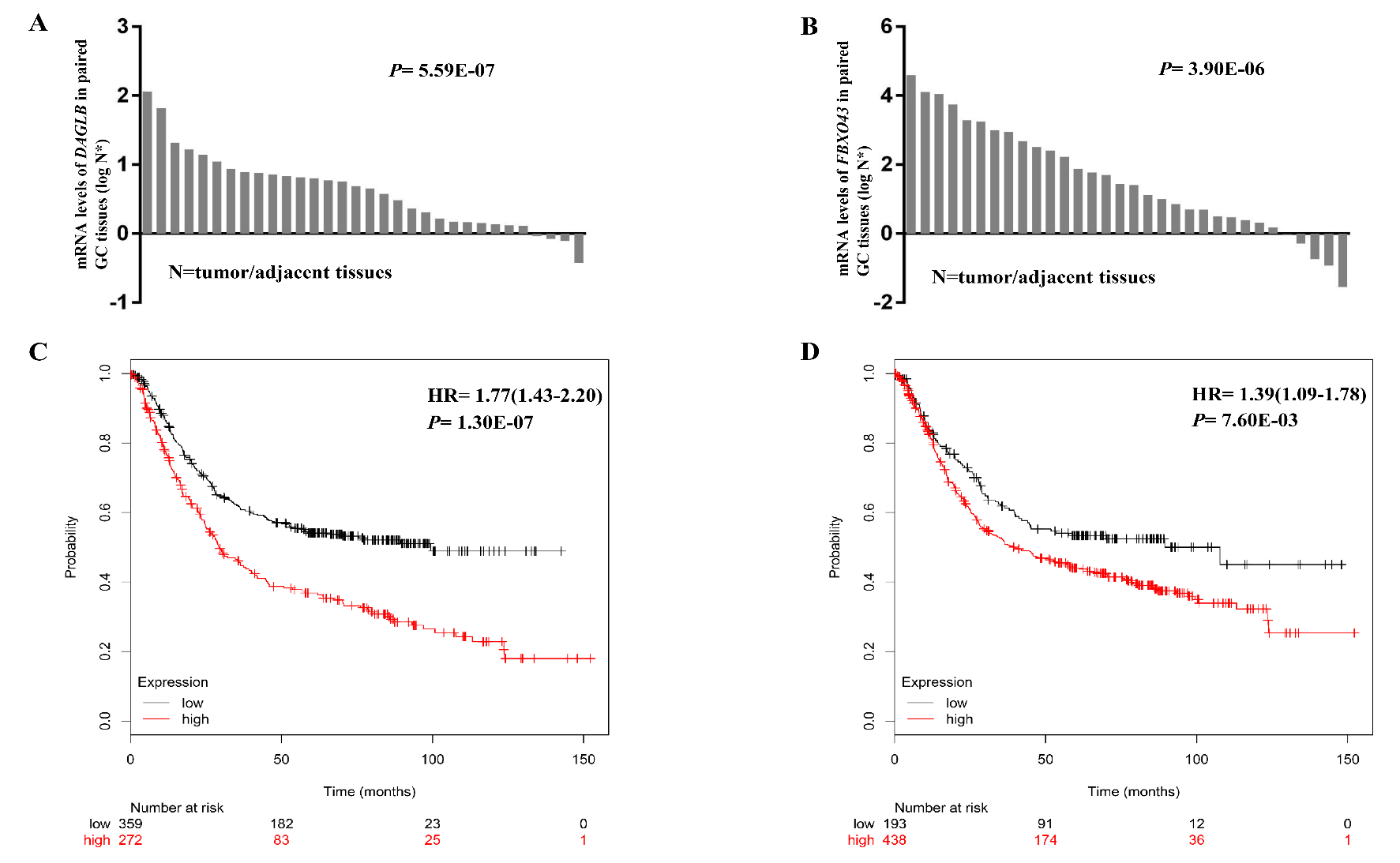


**Supplementary Figure S3.** *DAGLB* and *FBXO43* were remarkably overexpressed in tumors and associated with overall survival in GC patients. (A)(B) the mRNA levels of *DAGLB* and *FBXO43* in 32 GC tissues and matched adjacent normal tissues from TCGA database. (C)(D) Kaplan-Meier survival curve of patients with GC downloaded from online KM plotter database. Patients with higher *DAGLB* or *FBXO43* expression had a worse survival than those with lower expression (*P*=1.30×10^-7^, *P*=7.60×10^-3^, respectively).
